# An animal model of severe acute respiratory distress syndrome for translational research

**DOI:** 10.1186/s42826-025-00235-9

**Published:** 2025-01-24

**Authors:** Kuo-An Chu, Chia-Yu Lai, Yu-Hui Chen, Fu-Hsien Kuo, I.-Yuan Chen, You-Cheng Jiang, Ya-Ling Liu, Tsui-Ling Ko, Yu-Show Fu

**Affiliations:** 1 Division of Chest Medicine, Department of Internal Medicine, Kaohsiung Veterans General Hospital, Kaohsiung, Taiwan, ROC; 2 School of Medicine, College of Medicine, National Sun Yat-Sen University, No. 70, Lienhai Rd., Kaohsiung, Taiwan, ROC; 3 School of Nursing, Fooyin University, Kaohsiung, Taiwan, ROC; 4 Department of Nursing, Shu-Zen Junior College of Medicine and Management, Kaohsiung, Taiwan, ROC; 5 Institute of Anatomy and Cell Biology, School of Medicine, National Yang Ming Chiao Tung University, Taipei, Taiwan, ROC; 6 Department of Anatomy and Cell Biology, School of Medicine, National Yang Ming Chiao Tung University, No. 155, Sec. 2, Li-Nung Street, Taipei, Taiwan, ROC

**Keywords:** Acute respiratory distress syndrome (ARDS), Animal model, Acute lung injury, Arterial oxygen saturation (SpO_2_), Partial arterial pressure of oxygen (PaO_2_), Cytokine storm, Cell infiltration

## Abstract

**Background:**

Despite the fact that an increasing number of studies have focused on developing therapies for acute lung injury, managing acute respiratory distress syndrome (ARDS) remains a challenge in intensive care medicine. Whether the pathology of animal models with acute lung injury in prior studies differed from clinical symptoms of ARDS, resulting in questionable management for human ARDS. To evaluate precisely the therapeutic effect of transplanted stem cells or medications on acute lung injury, we developed an animal model of severe ARDS with lower lung function, capable of keeping the experimental animals survive with consistent reproducibility. Establishing this animal model could help develop the treatment of ARDS with higher efficiency.

**Results:**

In this approach, we intratracheally delivered bleomycin (BLM, 5 mg/rat) into rats’ left trachea via a needle connected with polyethylene tube, and simultaneously rotated the rats to the left side by 60 degrees. Within seven days after the injury, we found that arterial blood oxygen saturation (SpO_2_) significantly decreased to 83.7%, partial pressure of arterial oxygen (PaO_2_) markedly reduced to 65.3 mmHg, partial pressure of arterial carbon dioxide (PaCO_2_) amplified to 49.2 mmHg, and the respiratory rate increased over time. Morphologically, the surface of the left lung appeared uneven on Day 1, the alveoli of the left lung disappeared on Day 2, and the left lung shrank on Day 7. A histological examination revealed that considerable cell infiltration began on Day 1 and lasted until Day 7, with a larger area of cell infiltration. Serum levels of IL-5, IL-6, IFN-γ, MCP-1, MIP-2, G-CSF, and TNF-α substantially rose on Day 7.

**Conclusions:**

This modified approach for BLM-induced lung injury provided a severe, stable, and one-sided (left-lobe) ARDS animal model with consistent reproducibility. The physiological symptoms observed in this severe ARDS animal model are entirely consistent with the characteristics of clinical ARDS. The establishment of this ARDS animal model could help develop treatment for ARDS.

**Supplementary Information:**

The online version contains supplementary material available at 10.1186/s42826-025-00235-9.

## Background

Acute respiratory distress syndrome (ARDS) is a complicated disease that has many different pathogens, including one or more viruses, bacteria, fungal or mycobacterial species, and unknown factors. ARDS is a severe acute lung injury with devastating consequences. It is characterized by pulmonary inflammation, alveolar epithelial dysfunction, and microvascular permeability [1]. According to the Berlin definition for ARDS, an acute, diffuse, inflammatory lung injury, accompanied by low blood oxygen saturation (SpO2), respiratory failure, and other signs and symptoms, are seen within seven days after the patient was exposed to the risk factors [2, 3]. Patients with ARDS are often admitted to the intensive care unit due to respiratory and multiple organ failure, resulting in a tremendous burden on healthcare professionals and resources. ARDS has been widely known as a major health problem globally, carrying high morbidity and mortality [4–9]. Clinically, no medication, but only supportive therapies, is currently available for the management of ARDS. Thus, it is critical to develop treatments for ARDS.

Various animal models, such as hyperoxia, lipopolysaccharide, endotoxin, bacteria, BLM, etc., have been developed for acute lung injury [10–17]. These injury patterns cause lung inflammation and alveolar destruction and eventually lead to the sequela of pulmonary fibrosis. As previous studies demonstrated, there is significant diversity in pulmonary responses to LPS-induced acute lung injury between animal species and strains [18, 19]. This may reduce the correlation between animal test results and human trial outcomes. This is also why therapeutic effects on LPS-induced acute lung injury could not be replicated in patients with ARDS [20]. Moreover, a meta‑analysis of six studies involving 228 model rats suggested that higher‑quality and more rigorous studies are required to evaluate the potential utility of medicine or stem cells for treating BLM-induced lung injury and pulmonary fibrosis [17, 21]. There is a critical need to establish a rigorous ARDS animal model for medicine or stem cell development.

In this study, we modified the BLM-induced ARDS model to develop a severe, stable, and one-sided (left-lobe) ARDS with consistent reproducibility to evaluate precisely the therapeutic effect of medicine or stem cells on ARDS and simultaneously keep the experimental animals surviving.

## Methods

### Establishment of severe ARDS animal model

Male Sprague Dawley (S.D.) rats weighing 230–250 g were anesthetized via intraperitoneal injection of Zoletil 50 and Xylazine hydrochloride (Sigma 23076359). After deep anesthesia, 5 mg BLM dissolved in 200 μl sterile normal saline (1 unit activity/1 mg BLM, Nippon Kayaku Co., Ltd.) was intra-tracheally delivered into rats’ left trachea using a Hamilton syringe connected with a polyethylene tube (PE10, I.D. 0.28 mm, O.D. 0.61 mm. Length: 2.5 cm), as the rats were rotated to the left side by 60 degrees for 90 min to establish a severe, reproducible, left-lung damaged animal model.

### Experimental groups

**Fig. 1:**
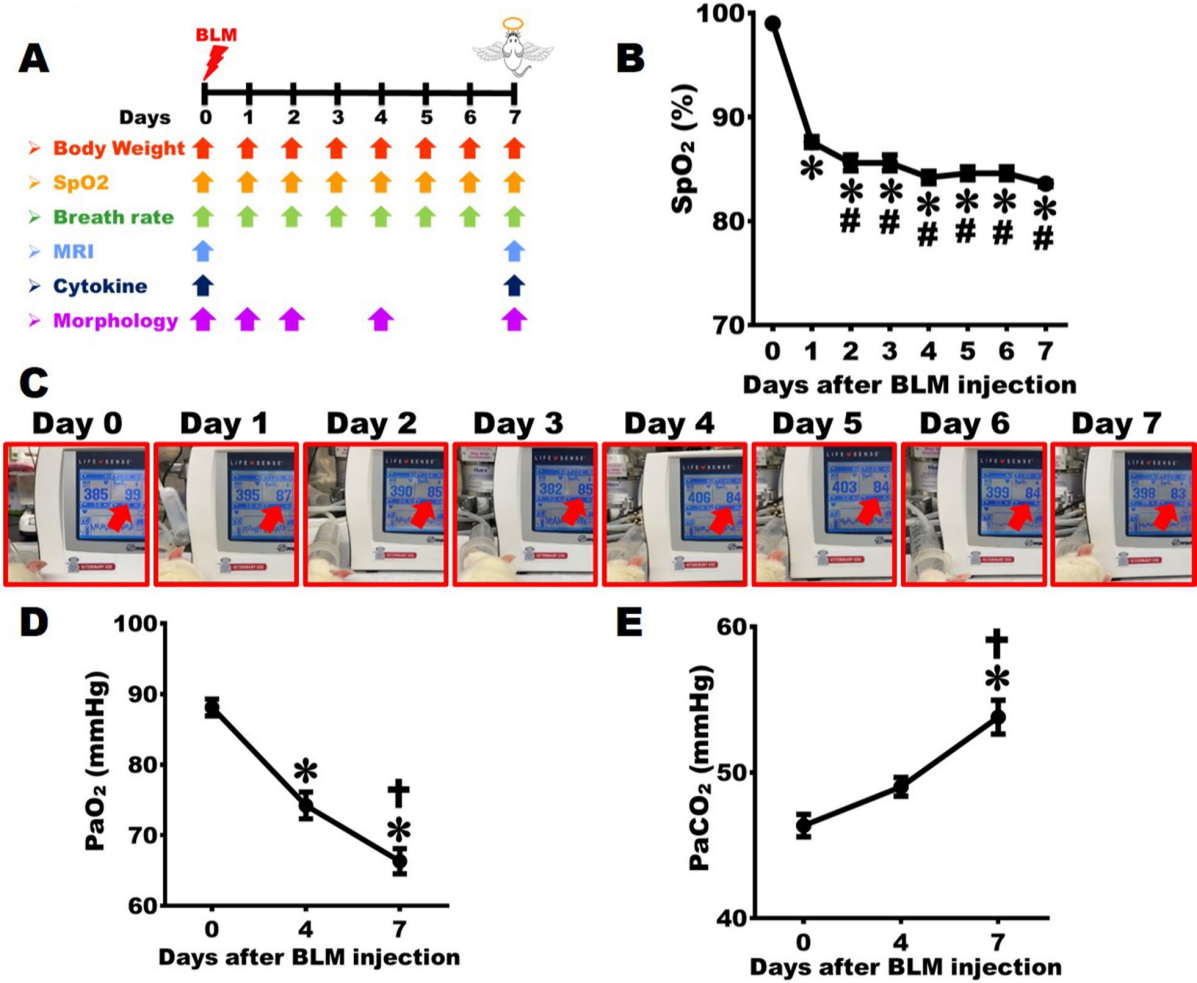
Establishment of severe ARDS animal model with low O_2_ level in the artery. **A** The study flowchart and experimental groups. BLM was injected into rats’ trachea on Day 0, and the animals were sacrificed on Day 7. **B** Daily changes of arterial SpO_2_ measured at rats’ hindlimbs; **C** the corresponding photographs from Day 0 to Day 7 after BLM damage. **D** Quantification of PaO_2_ at Day 0, Day 4, and Day 7, indicating that arterial PaO_2_ dropped to the lowest point on Day 7 post-BLM damage. **E** Quantification of PaCO_2_ at Day 0, Day 4, and Day 7, indicating that arterial PaCO_2_ surged to the peak on Day 7 post-BLM damage (n = 14). One-way ANOVA was applied for analysis. * *vs.* Day 0, p < 0.05. **#** *vs.* Day 1, p < 0.05. ☨ *vs.* Day 4, p < 0.05

The animals were randomly assigned into the following two groups: Group one, Normal group (n = 10): On Day 0, rats were intratracheally administered 200 μL of saline. Rats were sacrificed on Day 7. Group two, ARDS group (n = 32): On Day 0, rats received an intratracheal injection of 5 mg BLM. Rats were sacrificed on Day 1, 2, 4 and 7. The experimental flowchart is shown in Fig. 1A.

### Assessments of pulmonary function

#### Assessment of SpO_2_

Rats were anesthetized with isoflurane (Baxter 228–194). A pulse oximeter sensor (Pulse oximeter, NONIN LS1-10R) was clipped to the paws of their hind limbs to measure the arterial SpO_2_ weekly [22–24].

#### Assessment of PaO_2_ and PaCO_2_

Rats were anesthetized with isoflurane (Baxter 228–194). Arterial blood was drawn and mixed with 30 IU of heparin. Subsequently, a blood gas analyzer (Roche Cosbas b 221) was used to detect the arterial PaO_2_ and PaCO_2_.

#### Assessment of respiratory rate

Rats were placed in a closed cylinder-shaped detection chamber (emka Technologies, Whole body plethysmograph). The changes of breath flow within 15 min were recorded using the BIOPAC BSL 4.0 MP45, and the rats’ resting respiratory rates were quantified via the software AcqKnowledge.

### Magnetic resonance imaging (MRI)

Rat lung images were acquired via MRI (Bruker BioSpec 70/30) at the Instrumentation Center of National Taiwan University. The thoracic cavities of the rats were scanned from rostral to caudal and photographed horizontally every 1.5 mm until the whole thoracic cavity had been scanned. Since the first image obtained in each rat varied in position, the total number of slides of images in the horizontal plane varied in the range of 20 to 25.

The carina of the trachea was used as a landmark for image positioning. Five images contained the slice from the level of the carina, and four slices after the carina were summed for quantification of the black alveolar space to represent the left lung alveolar volume of the rats. Image-Pro Plus software was used for Image processing.

### Determination of cytokine /chemokine concentrations

The concentration of 26 cytokines were evaluated in the sera using the MILLIPLEX® RECYMAG65K27PMX Rat cytokine/chemokine magnetic bead panels kit (EMD Millipore, Burlington, MA, USA), including Eotaxin, Fractalkine, G-CSF, GM-CSF, interleukin-1 alpha (IL-1α), IL-5, IL-17A, IL-18, interferon gamma-induced protein-10 (IP-10), Leptin, lipopolysaccharide-inducible CXC chemokine (LIX), MCP-1, MIP-1α, MIP-2, normal T cell expressed and secreted (RANTES), tumor necrosis factor α (TNFα), VEGF, EGF, IFNγ, IL-1β, IL-2, IL-4, IL-6, IL-10, IL-12p70 and IL-13. In brief, the serum samples were mixed with antibody beads and were incubated in wells on a plate shaker at room temperature for 2 h. Well contents were then washed, and detection antibodies were added for 1-h incubation at room temperature. Samples were treated with streptavidin–phycoerythrin and incubated for another 30 min. After incubation, the supernatant was removed, plates washed, and beads re-suspended using assay buffer. Plates were subject to quantitate cytokine values for serum samples using a Luminex® 200TM reader.

### Embedding lung into paraffin blocks and tissue sectioning

Rats were euthanatized by anesthetic overdose with an intraperitoneal injection of Zoletil 50 and Xylazine hydrochloride (Sigma 23076359). Following fixation, the left lung was then embedded in paraffin.

The tissue block was shaped into trapezoidal pyramids, placed on a microtome and sectioned into 5-μm thick slices. Serial sagittal slices were sectioned starting from the outermost lateral side of the lung. The methodology of the slice collection procedure is shown in Additional file 1: Fig. S1. Subsequently, the slices were floated in 40- 45℃ warm water to smooth out wrinkles, placed on microscope slides, and then dried on a 50℃ hot plate.

### Hematoxylin and eosin (H&E) stain

Lung tissue sections were deparaffinized and immersed in hematoxylin solution (Muto Pure Chemicals Co., Ltd.; No. 3008–1) and then in eosin solution (Muto Pure Chemicals Co., Ltd.; No. 3200–2) (Additional file 1: Fig. S1, the first panel). The left lung volume and air space were determined via the summation of all H&E staining images and analyzed by Image-Pro software. The percentage of cell infiltration area represented the average of all H&E staining sections.

### Sirius red stain

After deparaffinization and rehydration, lung tissue sections were immersed in 0.1% sirius red (Sigma 2610–10-8) in picric acid for 10 min (Additional file 1: Fig. S1, the second panel). The percentage of collagen deposition was the average of red area ratio in all sirius red staining left lung sections (analyzed by Image-Pro software).

### Micro-computed tomography for fat mass and fat free mass

Rats were anesthetized using isoflurane on Day 7. Images were viewed using micro-computed tomography (micro-CT; MILabs, Utrecht, Netherlands). The range of images in length was 79.5 mm, and rats were imaged using 480 projections. The X-ray parameters were set at 0.48 mA and 50 kV. Finally, an image voxel was constructed using 80 μm. The images were analyzed using 3D-Slicer and separated into distinct body compositions based on tissue density in Hounsfield units (HU). The threshold for the fat tissue volume of the body below diaphragm was 350 to 150 HU, and the fat free mass volume (lean mass including muscles and organs) was 30 to 200 HU. The estimated tissue weight from micro-CT was determined using the volumetric density of fat mass (0.95 g/ cm^3^) and lean mass (1.05 g/ cm^3^). After obtaining the volumes in voxels per cm^3^ for fat tissue and fat free tissues, the amount of fat and lean mass were provided in grams.

### Protein mass of gastrocnemius

Gastrocnemius from Normal and ARDS groups was rinsed twice with PBS (4 °C) and lysed with lysis buffer (Merck, Millipore, 20–188). The protein mass of the tissue homogenates was determined by the Protein Assay Dye Reagent Concentrate (Bio-Rad, 5000006).

### Food intake assay

A sufficient amount of food pellets was placed on the cage, and the remaining food pellets were weighed and replaced with fresh food every day at 11:00 for seven days in a row.

### Measurement of serum biochemical parameters

The rat blood was centrifuged at 8000 rpm for 10 min, and the serum was collected and stored at − 20 °C. The serum levels of Triglyceride (TG), Cholesterol, LDL-C, and HDL-C were measured by LeZen Medical Technology Laboratory (Taipei). The level of Leptin was assessed using mouse/rat Leptin Quantikine enzyme-linked immunosorbent assay (ELISA) kits (Biotechne, R&D Systems).

### Statistical analysis

**Fig. 2:**
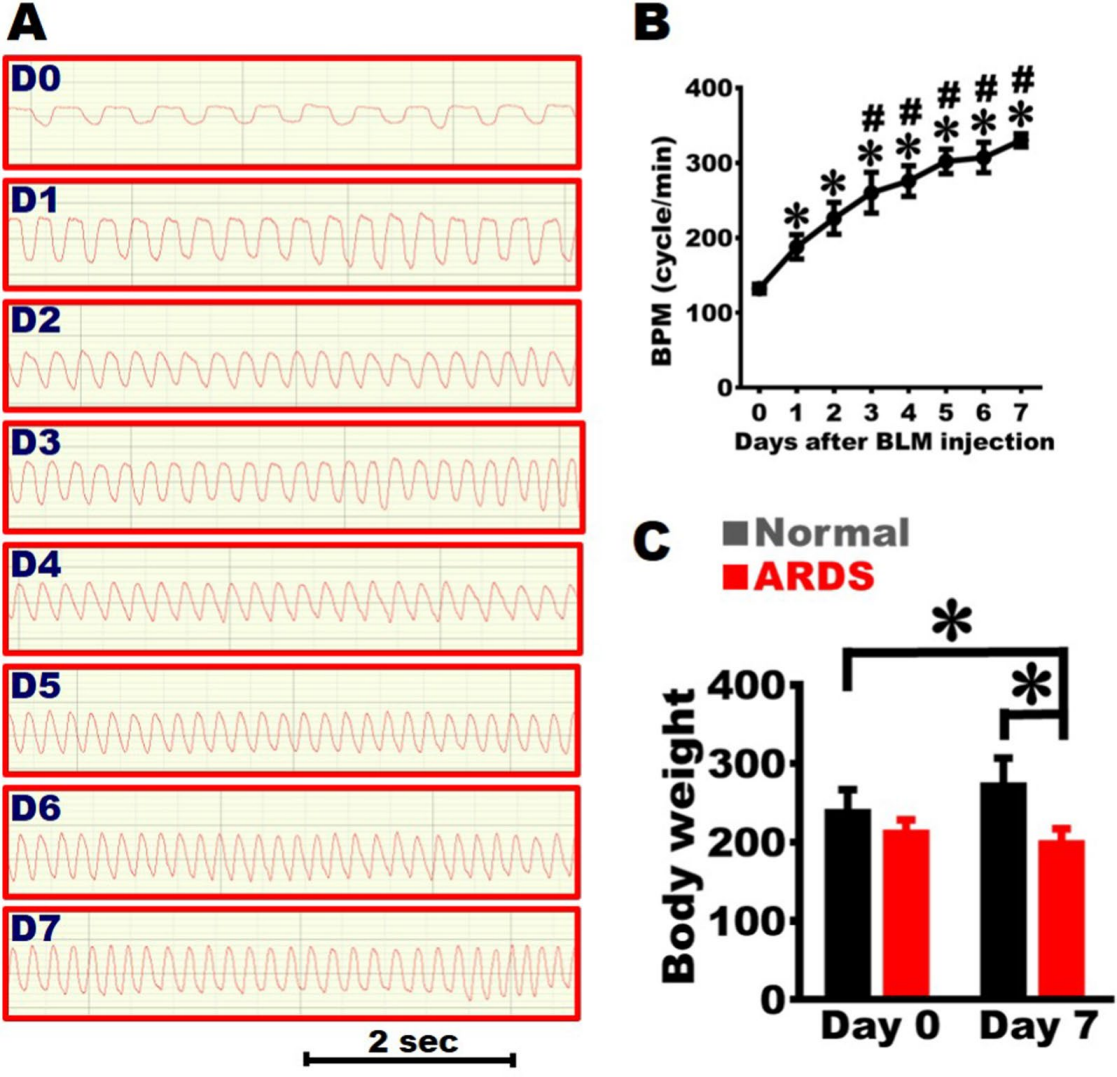
Establishment of severe ARDS animal model with rapid breathing. **A** The rats’ respiratory rates detected daily from Day 0 to Day 7 to quantify the respiratory frequency over 2 s. **B** Daily respiratory frequency from Day 0 to Day 7 after BLM damage, revealing that the frequency reached the peak on Day 7 post-BLM damage. **C** Body weight at day 0 and day 7, indicating that the body weights decreased on Day 7 post-BLM damage (n = 14 for ARDS, n = 6 for Normal group). One-way ANOVA was applied for analysis. * *vs.* Day 0 or Normal group, p < 0.05. **#** *vs.* Day 1, p < 0.05

**Fig. 3:**
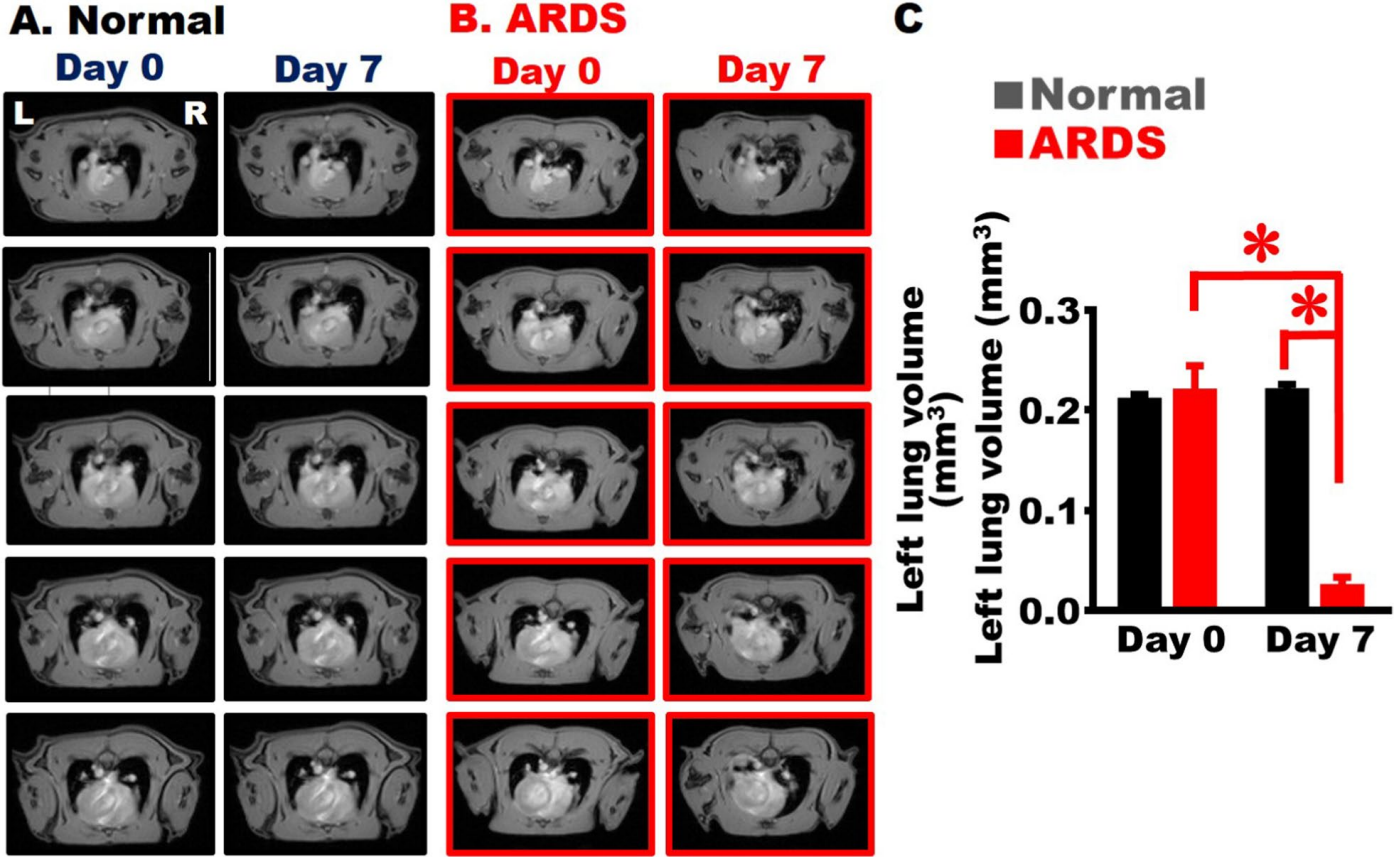
Establishment of severe ARDS animal model with loss of alveolar space. Five MRI scans of rats’ thoracic cavities in the Normal (**A**) vs. ARDS (**B**) groups, with carina of the trachea as a benchmark for image positioning; space occupied by alveoli in the thoracic cavity appears as black. “L” and “R” show the left side and the right side of the body, respectively. **C** Black alveolar spaces, averaged over five MRI scans, for each rat indicated that, compared with the result on Day 0, the alveolar volume decreased significantly on Day 7 after BLM injury (n = 4 in each group). One-way ANOVA was applied for analysis. *, p < 0.05

**Fig. 4:**
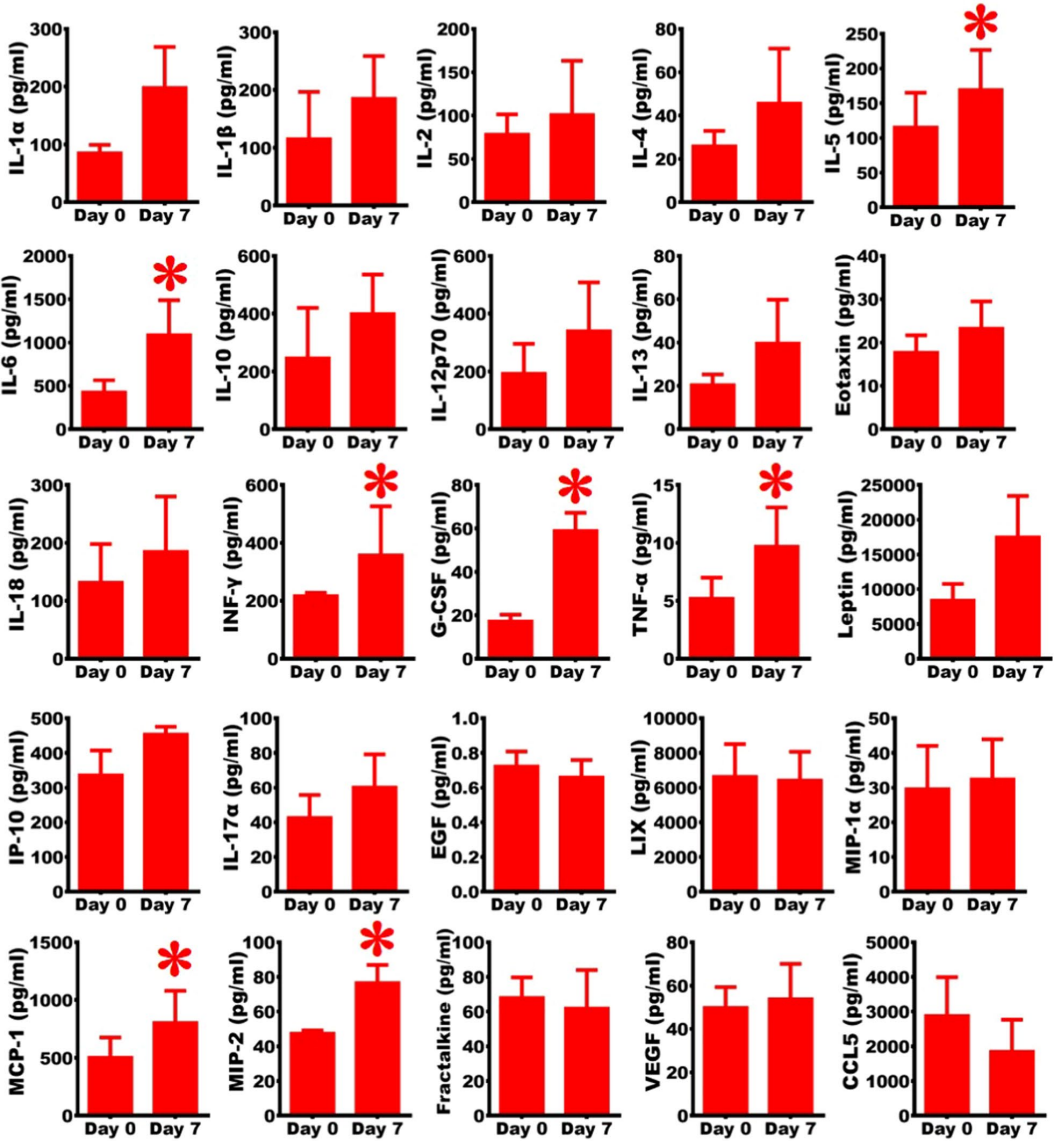
Establishment of severe ARDS animal model with inflammatory storm. A comparison of the pro-inflammatory and inflammatory cytokines at Day 0 vs. Day 7 after BLM damage, indicating the increment in the cytokines on Day 7 (n = 3). Student's t-test was applied for analysis. *, *vs.* Day 0, p < 0.05

**Fig. 5:**
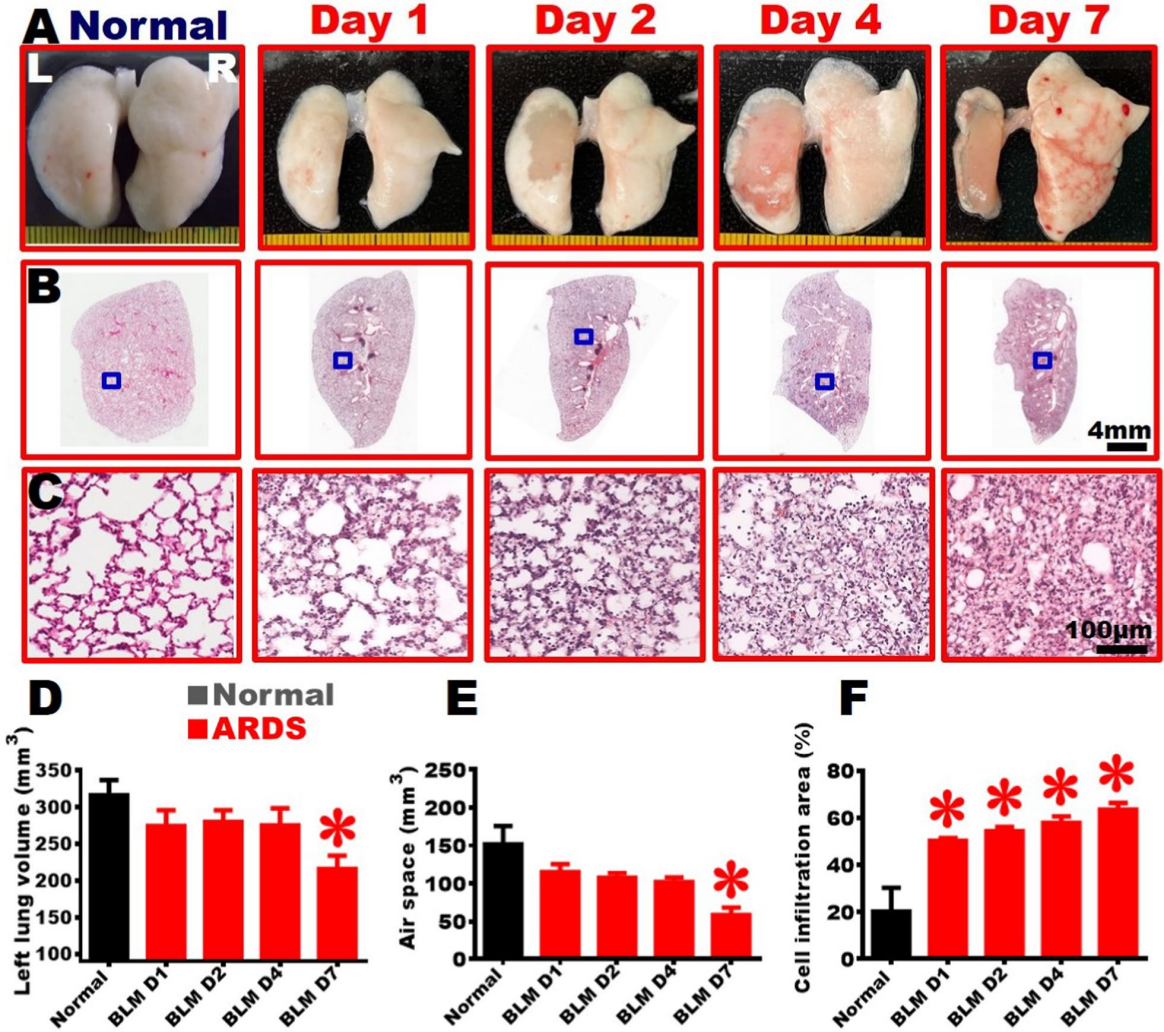
Establishment of severe ARDS animal model with pathological changes in morphology. **A** The overall appearance of the lung on different days (Day 0, 1, 2, 4, and 7) following BLM damage. **B** Low magnification images of the left-lung tissue sections stained with H&E; **C** High magnification images of the central area (blue squares in **B**) of the lung. Following BLM damage, the central area was infiltrated with a large number of cells, and the alveoli almost disappeared. Averaged over sections stained with H&E (**D**: total volume of left lung; **E**: air space of left lung; **F**: percentage of cell infiltration area), showing the volume of left lung gradually decreased, the number of alveoli decreased, and the percentage of cell-infiltration area increased rapidly (n = 6 in each time point). One-way ANOVA was applied for analysis. *, *vs.* Day 0, p < 0.05

**Fig. 6:**
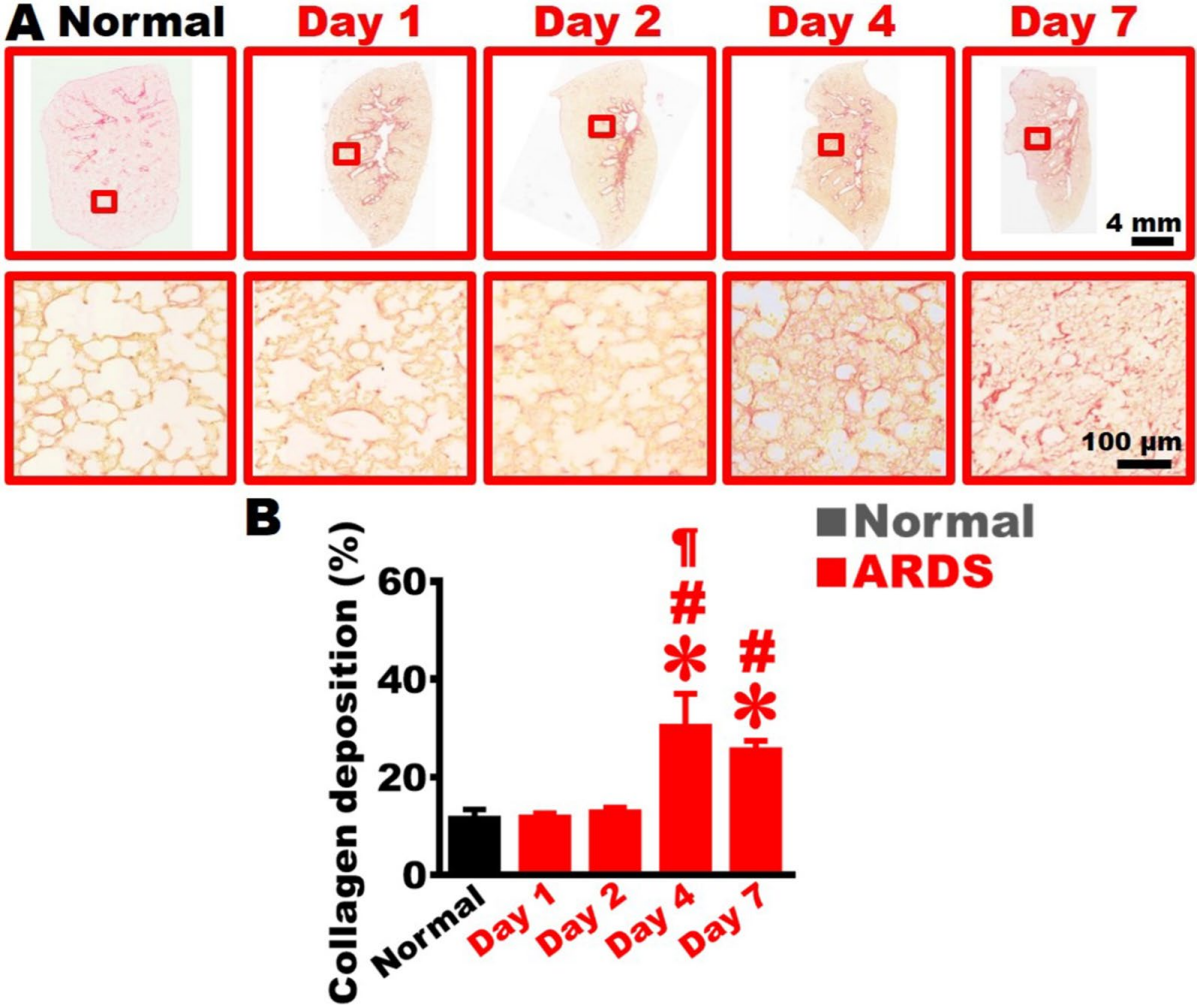
Establishment of severe ARDS animal model with collagen accumulation. **A** The left-lung tissue sections stained with Sirius red are displayed at low and high magnifications (red squares) from Day 1 to Day 7, the red area indicates the presence of fibrosis. **B** Quantifications of the area of collagen stained with Sirius red indicated collagen deposition increased since Day 4 post BLM damage (n = 6 in each time point). One-way ANOVA was applied for analysis. * *vs.* Day 0, p < 0.05. **#** *vs.* Day 1, p < 0.05. **¶** *vs.* Day 2, p < 0.05

**Fig. 7:**
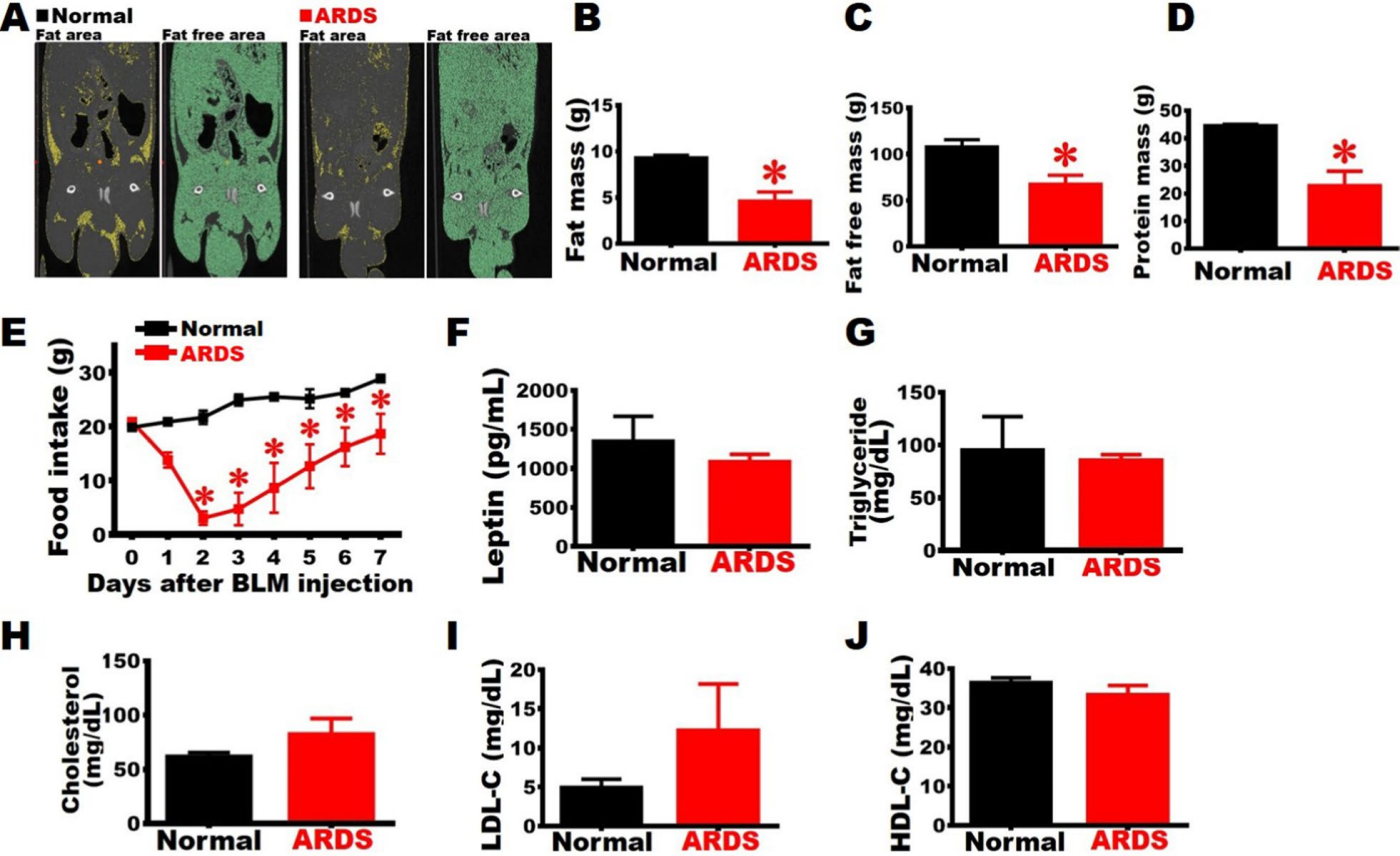
Establishment of severe ARDS animal model with fat mass decrease. **A** Representative images taken by micro-CT of the Normal and ARDS groups on Day 7. The yellow regions in left panel indicate the fat distribution areas, while green regions in the right panel represent the non-fat distribution areas. The fat mass and non-fat mass were estimated using the volume in the CT scans, and the results demonstrated that rats in the ARDS group had considerably lower fat mass and non-fat mass (**B** and **C**). The protein content in the gastrocnemius and the food intake of rats with ARDS decreases considerably (**D** and **E**). The Leptin, Triglyceride, Cholesterol, LDL-C, and HDL-C levels in the sera of rats with ARDS are similar to those in the Normal group (**F**-**J**). N = 3. Student's t-test was applied for analysis. * *vs.* Normal group at the same day, p < 0.05

All experimental data are presented in Mean ± SEM (standard error of the mean). Student's t-test or One-way analysis of variance (ANOVA) followed by Turkey’s test for multiple comparisons were used. One-way ANOVA was applied for analysis the SpO_2_, O_2_, and CO_2_ in Fig. 1, BPM and body weight in Fig. 2, images of MRI in Fig. 3, histological morphology in Fig. 5 and Fig. 6. Student's t-test was applied for analysis the cytokines of serum in Fig. 4 and Fig. 7. A p-value of less than 0.05 was considered statistically significant.

## Results

### Low O_2_ and high CO_2_ levels in the severe ARDS rats

The rats’ oxygenation index was first assessed by a pulse oximeter daily within 7 days after BLM damage, with the paws of their hind limbs clipped to measure the efficiency of pulmonary gas exchange. On Day 0 (before injury), the arterial SpO_2_ was generally maintained at 97.9 ± 0.2%. The SpO_2_ statistically dropped to 87.5 ± 0.2% on Day 1, and significantly declined to 85.1 ± 0.3% on Day 2; it maintained around 84.8 ± 0.4% to 83.7 ± 0.3% from Day 3 to Day 7 (Figs. 1B- 1C). The PaO_2_ and PaCO_2_ in the tail artery were detected to further estimate rats’ lung function. The PaO_2_ was 89.2 ± 2.9 mmHg on Day 0 (before injury); it significantly reduced to 71.8 ± 3.3 mmHg on Day 4 and then substantially diminished to 65.3 ± 3.3 mmHg on Day 7 (Fig. 1D). Before injury (on Day 0), the PaCO_2_ was 45.3 ± 1.2 mmHg; it noticeably elevated to 49.2 ± 1.9 mmHg on Day 7 (Fig. 1E). These results showed that the oxygenation index decreased significantly within seven days after BLM injury.

### Rapid breathing in the severe ARDS rats

Breaths per minute (BPM) were daily counted to estimate lung function (Fig. 2A). Respiratory rate was 129.9 ± 5.8 cycles/min on Day 0 (before injury), increased to 187.1 ± 8.4 cycles/min on Day 1 and then significantly elevated to 207.8 ± 12.4 cycles/min on Day 2. The respiratory rate continued to increase since then; it elevated to 260.1 ± 12.0 cycles/min on Day 4 and raised to 328.7 ± 6.8 cycles/min on Day 7 (Figs. 2A-2B). These findings indicated that BLM damage caused a loss in lung function, hypoxemia, and shortness of breath within seven days of the injury.

### Stagnant body weight in the severe ARDS rats

Body weight of rats was 225.8 ± 6.9 g on Day 0 and elevated to 265.9 ± 19.1 g on Day 7 in the Normal group, indicating that their body weight increased over time. After BLM injury, the body weight was 211.2 ± 5.5 g on Day 0 and stalled around 197.7 ± 6.2 g on Day 7. Their weights were significantly lower than those in the Normal group (Fig. 2C). The results suggested that BLM damage caused a stagnation in body weight within seven days of the injury.

### Lost alveolar space in the severe ARDS rats

In the horizontal MRI at the level of the trachea carina, the black signals occupying the thoracic cavity are the symbols of the alveoli, whereas the white signals represent consolidated tissues. Five images contained the first slice from the level of the carina, and four slices after the carina were shown (Fig. 3A–3B). Alveoli existed in both lungs of the Normal group, and black signals were observed predominantly from Day 0 to Day 7 (Fig. 3A). White signals appeared in the left lungs because of the inflammatory responses and cell infiltration occurred on Day 7 after BLM injury (Fig. 3B). Five images were summed for quantification of the black alveolar space to represent the left lung alveolar volume of the rats, revealing that the alveolar volume in the left lungs reduced significantly within seven days after damage (Fig. 3C).

### Intense immune response in the severe ARDS rats

The arterial serums were obtained to analyze the cytokines. The results showed that the majority of the pro-inflammatory or inflammatory factors were slightly increase on Day 7. Among them, IL-5, IL-6, IFN-γ, MCP-1, MIP-2, G-CSF, and TNF-α significantly increased compared with those in Day 0 (before injury) (Fig. 4). The results revealed that BLM damage caused cytokine storms within seven days after the injury.

### Diffuse cell infiltration in the lung of the severe ARDS rats

The overall appearance of the lung revealed that the right and left lungs of the Normal group were intact and smooth. On Day 1 following injury, the left lung showed an irregular and uneven surface. On Day 2, the central region of the left lung had lost some of the white alveoli. On Day 4, no signs of the white alveoli were observed in the central region of the left lung; alveoli were only presented on the margin of the left lung. On Day 7, the left lung markedly shrunk, and only a thin layer of alveoli was seen on the margin of the left lung (Fig. 5A). Subsequently, the left lung tissues were subjected to H&E staining to examine the detailed morphological changes with low and high magnifications (Fig. 5B-5C). The results revealed the presence of alveolar tissues in the normal left lung. The connective tissues were predominantly found around the bronchus, with only a few identified between alveoli. On Day 1 post-injury, cell infiltration started to appear in the left lung and between the alveoli. From Day 2 to Day 7, the condition of cell infiltration worsened, and the alveoli in the central region almost disappeared; only some alveoli were seen in the periphery of the left lung (Fig. 5B-5C). Moreover, we summed up the results from all left-lung sections stained with HE staining to quantify the total volume of the left lung. On Day 1, the volume of the left lung was 280.1 ± 12.9 mm^3^. On Day 7, the volume the left lung markedly decreased to 194.5 ± 6.7 mm^3^ as compared to those of Normal, Day 1 and Day 2; in addition, the air space also significantly decreased (Fig. 5D-5E). The cell-infiltrating consolidated tissues significantly increased to around 53.3 ± 2.7% of the volume of the left lung from Day 1 to Day 4. The percentage of the cell-infiltration area filled up to 68.2 ± 2.2% on Day 7 (Fig. 5F). The results showed that BLM damage led to cell infiltration, alveolar disappearance, and lung shrinkage within seven days after the injury.

### Increased collagen accumulation in the lung of the severe ARDS rats

The left lung sections were stained with Sirius red to label collagen and mages from low to high magnifications were examined (Fig. 6A). In the left lung of the Normal group, collagen mainly appeared around the bronchus and vessels and the percentage of collagen deposition was around 13.4 ± 1.3. In the left lung of the BLM damage, the red area representing collagen significantly increased to 30.3 ± 6.7% and 25.5 ± 1.9% on Day 4 and Day 7, respectively (Fig. 6B). The results revealed that BLM damage caused an elevation in collagen deposition in the lung within seven days.

### Physiological parameters changed in the severe ARDS rats

To determine whether ARDS alters lipid metabolism, the body fat mass below the diaphragm was measured by micro-CT (Fig. 7A). The images showed that rats in the ARDS group were significantly thinner than those in the Normal group. The rats in ARDS group had considerably reduced fat mass and fat free mass (i.e. muscles and organs) than those in the Normal group on Day 7 (Fig. 7B-7C). Furthermore, the total amount of protein in the gastrocnemius was significantly lower in the ARDS group compared to that of the Normal group (Fig. 7D). Daily food consumption measurements revealed that, beginning on the second day after BLM damage, rats in the ARDS group ate considerably less than those in the Normal group. Although food intake grew day by day, there was a substantial difference between the ARDS and Normal groups at the same day (Fig. 7E). Leptin may regulate food intake, hence we analyzed its content in serum. The results revealed that no statistical difference in Leptin concentration between rats in the ARDS and Normal groups (Fig. 7F). In addition, rats in the ARDS and Normal groups did not differ in their Triglyceride, Cholesterol, LDL-C, or HDL-C levels (Fig. 7G-7J). We suggest that BLM injury induces acute lung inflammation, and that the rats' physical illness causes a temporary dip in food intake, resulting in a decrease in body weight and fat mass. However, there has been no significant change in the biomarkers of lipid metabolism in the serum.

## Discussion

In this study, we injected BLM intratracheally into male S.D. rats to establish a unique left-lobe-dominated ARDS animal model accompanied by SpO_2_, shortness of breath, cell infiltration in lung and immune storm on Day 7 post- BLM damage.

Although accumulated studies have focused on developing treatments for acute lung injury. To date, ARDS is still a difficult challenge in clinical medicine. We suggest that it is caused by a discrepancy between the lung function and pathology of the animal model in the previous studies and the clinical symptoms of ARDS. To achieve a reproducible and consistent effect on pathological change and to ensure a better quality of life and higher survival rate of the rats, BLM was injected into the rat’s left trachea. The rat was then rotated toward its left side by 60 degrees for 90 min to establish a severe left-lung ARDS animal model to precisely evaluate the effect of medication or stem cell therapy. This animal model offers three key advantages: First, the rat could survive with its right lung. Second, the left lung has apparent changes in gross appearance. Third, with around 50% of the air inhaled enters the left lung, the severely damaged left lung leads to the fact that 50% of the exchange of oxygen and carbon dioxide is obstructed, and the pulse oximeter could be applied to detect the hypoxemia. Hence, the animal model could be employed to accurately assess the effects of medication or stem cell administrations. According to Berlin’s definition**:** (1) ARDS is a diffuse process reflected in bilateral radiographic abnormalities; (2) Severely impaired gas exchange is reflected by the ratio of measured PaO_2_ to the fraction of inspired oxygen (FiO_2_); (3) The radiographic and physiologic aberrations are not a primary consequence of heart failure [2]. In the present study, the rat’s arterial SpO_2_ decreased to around 83%, the arterial PaO_2_ reduced to 65 mmHg, the respiratory rate increased to 329 cycles/min, substantial cell infiltration appeared among alveolar tissues, and alveoli disappeared on Day 7 after BLM injury. These signs were close to the characteristics of clinical ARDS. Our previous studies used the same injury method to establish the animal model of lung fibrosis. The BLM injury was tracked for 49 days. The results showed that the left lung shrank, the alveoli disappeared, and collagen accumulation peaked on Day 49. These findings revealed that this severe ARDS animal model leads to significant lung fibrosis [22–24].

Excessive production of proinflammatory and inflammatory cytokines leads to ARDS exacerbation and widespread tissue damage resulting in multi-organ failure and death. Accumulating evidence suggests that mortality in patients with ARDS could be linked to the presence of the so-called cytokine storm [25–29]. Previous studies reported increased levels of IL-10, IL-6, and TNF-α in severe COVID-19 patients [29, 30]. Other studies documented elevated levels of IL-1β, IL-7, IL-8, IL-9, IL-10, FGF, G-CSF, GM-CSF, IFN-γ, IP-10, MCP-1, MIP-1A, MIP1-B, PDGF, TNF-α, and VEGF in both patients with ARDS admitted to the ICU and non-ICU patients compared to healthy adults [27, 28]. Their results are comparable to our findings. In this study, we observed that IL-5, IL-6, IFN-γ, MCP-1, MIP-2, G-CSF, and TNF-α in arterial serums significantly increased on Day 7 compared with those before injury. The results revealed that cytokine storms were triggered by a severe ARDS animal model induced by BLM.

## Conclusions

The present study modified the BLM-induced lung injury animal model to develop a severe, stable, one-sided (left-lobe) ARDS with consistent reproducibility. The physiological symptoms of this severe ARDS animal model are fully consistent with the pathological definition of clinical ARDS. Most importantly, the severity of the pathological situation remains consistent each time. Establishing this ARDS animal model could provide greater accuracy for future drug development to treat acute lung injury, benefiting patients with acute lung injury or ARDS.

## Supplementary Information

Additional file 1.

## Data Availability

The corresponding author, YSFu, will make all the data behind the study's conclusions available to the public under reasonable request.

## Abbreviations

ARDS: Acute respiratory distress syndrome
BLM: Bleomycin
BPM: Breaths per minute
H&E stain: Hematoxylin and eosin (H&E) stain
MRI: Magnetic resonance imaging
Micro-CT: Micro-computed tomography
PaO_2_: Partial pressure of arterial oxygen
PaCO_2_: Partial pressure of arterial carbon dioxide
SpO_2_: Arterial blood oxygen saturation
