# Supplementary material for: An animal model of severe acute respiratory distress syndrome for translational research
Source: Lab Anim Res. 2025 Jan 24;41:4. doi: 10.1186/s42826-025-00235-9 (PMC11758736; doi:10.1186/s42826-025-00235-9)
Supplement: Supplementary file 1 — Additional file 1. [file 42826_2025_235_MOESM1_ESM.docx]

**Supplemental Figure 1. Sectioning of lung tissues and slice allocation for staining**

A consecutive of 10 slices, numbered 1 to 10, were placed on different slides for different immunostainings. Taking the Normal group as an example, lung slices numbered 1 to 10 were placed on slides labeled as1-1, 1-2, 1-3…… to 1-10. Slices numbered 11 to 30 were discarded (a total of 20 slices). Likewise, slices numbered 31 to 40 were placed on the second place of slides from 1-1, 1-2, 1-3…… to 1-10. The next 20 slices were also discarded. The same procedures were repeated until the entire lung tissue was completely sectioned. Column #I (1-1, 1-2, 1-3, 1-4……1-10) represent the outermost region of each lobe in the left lung; Column # 2 (2-1, 2-2, 2-3, 11-4……2-10) represent the region close to hilum in the left lung. Lung slices on slides 1-1, 2-1, 3-1, 4-1, 5-1 ….and 11-1 were all subjected to H&E statin to assess the histopathology of the tissue; lung slices 1-2, 2-2, 3-2, 4-2, 5-2 ….and 11-2 were stained with Sirius red for evaluation of tissue fibrosis; The remaining slides were preserved as spares. The number of lung slices obtained varied in different groups (in the range of 330 to 880 slices).
